# Succinate Dehydrogenase B Subunit Immunohistochemical Expression Predicts Aggressiveness in Well Differentiated Neuroendocrine Tumors of the Ileum

**DOI:** 10.3390/cancers4030808

**Published:** 2012-08-16

**Authors:** Massimo Milione, Sara Pusceddu, Patrizia Gasparini, Flavia Melotti, Patrick Maisonneuve, Vincenzo Mazzaferro, Filippo G. de Braud, Giuseppe Pelosi

**Affiliations:** 1 Department of Pathology and Laboratory Medicine, Fondazione IRCCS Istituto Nazionale dei Tumori, Milan I-20133, Italy; 2 Department of Medical Oncology, Fondazione IRCCS Istituto Nazionale dei Tumori, Milan I-20133, Italy; 3 Molecular Cytogenetics Unit, Fondazione IRCCS Istituto Nazionale dei Tumori, Milan I-20133, Italy; 4 Division of Epidemiology and Biostatistics, European Institute of Oncology, Milan 20141, Italy; 5 Division of Gastrointestinal Surgery and Liver Transplantation, Fondazione IRCCS Istituto Nazionale dei Tumori, Milan I-20133, Italy; 6 Department of Medicine, Surgery and Dentistry, Università degli Studi, Facoltà di Medicina, Milan 20122, Italy

**Keywords:** midgut, neuroendocrine tumors, SDHB, immunohistochemistry, Ki-67 antigen

## Abstract

Immunohistochemical loss of the succinate dehydrogenase subunit B (SDHB) has recently been reported as a surrogate biomarker of malignancy in sporadic and familial pheocromocytomas and paragangliomas through the activation of hypoxia pathways. However, data on the prevalence and the clinical implications of SDHB immunoreactivity in ileal neuroendocrine tumors are still lacking. Thirty-one consecutive, advanced primary midgut neuroendocrine tumors and related lymph node or liver metastases from 24 males and seven females were immunohistochemically assessed for SDHB. All patients were G1 tumors (Ki-67 labeling index ≤2%). SDHB immunohistochemistry results were expressed as immunostaining intensity and scored as low or strong according to the internal control represented by normal intestinal cells. Strong positivity for SDHB, with granular cytoplasmatic reactivity, was found in 77% of primary tumors (T), whilst low SDHB expression was detected in 90% of metastases (M). The combined analysis (T+M) confirmed the loss of SDHB expression in 82% of metastases compared to 18% of primary tumors. SDHB expression was inversely correlated with Ki-67 labeling index, which accounted for 1.54% in metastastic sites and 0.7% in primary tumors. A correlation between SDHB expression loss, increased Ki-67 labeling index and biological aggressiveness was shown in advanced midgut neuroendocrine tumors, suggesting a role of tumor suppressor gene.

## 1. Introduction

Ileal neuroendocrine tumors (INETs) are the most common type of neuroendocrine neoplasms in the gastrointestinal tract, with a male prevalence and a median age at the time of diagnosis of 66 years. They are mainly composed of enterochromaffin cells (EC) producing serotonin and substance P [1,2]. A distinctive feature of INETs, especially when involving the liver, is their capability of causing distinct clinical syndromes [1,3,4], which can be faithfully monitored measuring the relevant hormones in the bloodstream. Surgical resection can be curative in early stage patients [5,6], but most of them present with liver involvement at the time of diagnosis, so tumor grading and staging according to WHO/AJCC/ENET criteria are likely to play the most important role in the prognostic and therapeutic assessment of INETs [6]. Accordingly, neuroendocrine carcinomas, which show high proliferative activity as reflected by Ki67 labeling index (LI) over 20%, are treated with cisplatin-etoposide combination chemotherapy similarly to small cell lung cancer (SCLC) [7,8], whereas most ileal neuroendocrine tumors are slowly growing neoplasms, which mainly depend on angiogenesis for their maintenance and growth [9,10,11,12,13,14]. Hence the search for new markers capable of getting new insights into the biological properties of INETs may be clinically warranted.

The succinate dehydrogenase (SDH) enzyme (also known as succinate ubiquinone oxydoreductase) is a highly conserved heterotetrameric protein, with SDHA and SDHB functioning as catalytic subunits, which protrudes into the mitochondrial matrix and is anchored to the inner membrane by means of SDHC and SDHD subunits, the latter also providing the binding site for ubiquinone [15,16,17,18,19,20,21,22,23,24,25,26,27,28,29,30,31,32,33,34,35,36,37,38,39,40]. SDHB is normally ubiquitously expressed with granular cytoplasmic immunostaining reflecting its mitochondrial location [41,42]. It is also been shown that silencing SDHB expression induces tumor-like phenotypic traits in cell cultures [43], and that the loss of any subunit protein, especially B, leads to the loss of SDH expression due to destabilization of its complex [20,44]. However, data on the prevalence of SDHB in INETs and its implications on tumor differentiation and prognosis are still lacking, to the best of our knowledge.

This study was aimed at evaluating the distribution of SDHB by immunohistochemistry (IHC) in 31 INETs and corresponding lymph node or liver metastases in order to explore its diagnostic and prognostic implications.

## 2. Results and Discussion

There were no differences between functioning (FT) and nonfunctioning (NFT) groups in age, gender, clinical outcome and medical treatment. The only significant association was the greater liver tumor load (*p* = 0.0000026) and the increased basal chromogranin A (CgA) serum level (*p =* 0.0113) in the NFT group (Table 1).

**Table 1:** Demographic and clinicopathologic information on the 31 INETs patients under evaluation.

Variable		NFT	FT	p Value
**Age (Years)**	<50	8	4	0.106
	51–70	7	9	
	>70	0	3	
**Gender**	Male	13	12	0.653
	Female	2	4	
**Outcome**	DOD	5	8	
	AW	0	0	0.3480
	AWD	7	3	
	A.NED	3	3	
**Medical Treatment**	SMS	12	13	
	CT	2	3	0.653
	SMS+CT	0	0	
	NO	1	0	
**Liver Tumor Load**	H1	13	1	0.0000026
	H2	1	1	
	H3	1	14	
**Basal CgA ng/mL**	<200	10	3	0.0113
	>200	5	13	

F, female; M, male; DOD, died of disease; AW, alive and well; AWD, alive with disease; A.NED, not evidence of disease; H1, liver involvement <25%; H2, liver involvement between 25 and 50%; H3, liver involvement >50%; INETs, ileal neuroendocrine tumors; SMS, somatostatin analogues; CT, chemotheraphy; CgA, chromogranin A.

**Figure 1:**
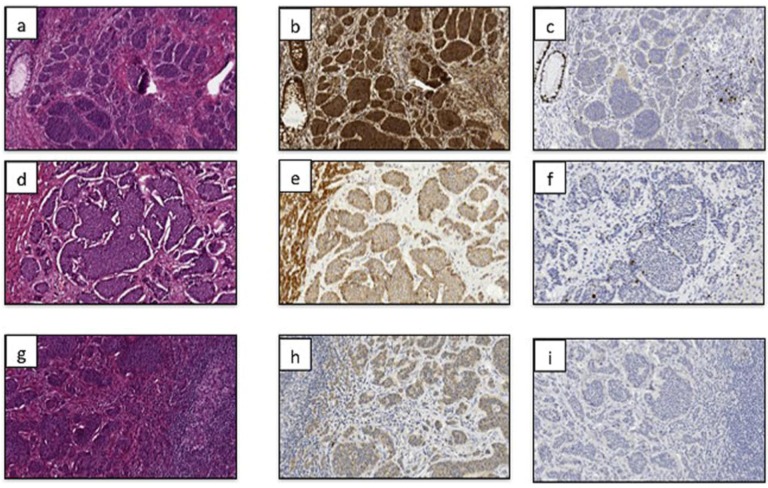
Representative pictures of primary neuroendocrine tumors of the ileum stained with hematoxylin and eosin (**a**,**d**,**g**), and SDHB (**b**,**e**,**h**) and Ki-67 antigen (**c**,**f**,**i**) immunohistochemistry are shown in primary lesions (**a–c**), and lymph node (**d–f**) or liver metastases (**g–i**). All tumor cells exhibited granular staining due to the mitochondrial accumulation of immunreaction product. A clear gradient of SDHB expression loss can be easily appreciated going from primary tumors (panel “**b**”) to lymph node (panel “**e**”) and liver (panel “**h**”) metastases. The normal epithelial and mesenchimal cells present in the tumor tissue samples served as internal positive controls (for example, the intestinal gland structures detectable on the right side of the panel “**b**” show a clear and specific decoration for the marker under assessment).

SDHB immunoreactivity was found in tumor cells of all cases under assessment, but there was a significant relationship between SDHB intensity and percentage of immunoreactive cells (test for trend) (Table 2). Representative pictures of SDHB and Ki-67 antigen immunoreactivity are depicted in Figure 1. In particular, the more the intensity of immunoreactivity, the more the percentage of positive cells. The percentage of tumor cells was associated significantly with the site of tumors and Ki67 LI, since primary lesions bearing a proliferative activity ≤1.3% showed over 50% SDHB immunoreactive tumor cells (Table 3). Likewise, significant associations were found between the site of tumors (*p* < 0.0001) or Ki-67 LI (*p* < 0.0001) and SDHB immunostaining intensity (Table 3). No significant associations were found with age, gender, type of therapy, presence of clinical syndrome, and CgA level.

**Table 2:** Relationship between SDH intensity and percentage of positive tumor cells

	All Measures	SDHB intensity			*p* Value
		1	2	3	
All measures	39	19	9	11	
SDHB expression					
1–25%	5	4	0	1	
26–50%	9	6	2	1	
51–75%	15	8	4	3	0.076 (Fisher exact)
76–100%	10	1	3	6	0.007 (trend)

**Table 3:** Distribution of tumor site (primary *vs*. metastasis) and Ki-67 labeling index according to SDHB expression (percentage of positive tumor cells) and immunstaining intensity.

SDHBExpression	SITE			Ki-67 labeling index		
	Primary Tumor	Metastases	*p* Value	≤1.3%	≥1.3%	*p* Value
1–25%	2	3		1	4	
26–50%	2	7	0.013 (trend)	3	6	0.038
51–75%	7	8		8	7	
76–100%	9	1		7	3	
**SDHB Intensity**						
1+	1	18		2	17	
2+	8	1	<0.0001(trend)	7	2	<0.0001(trend)
3+	11	0		10	1	

Ki-67 labeling index: 1.3% represented the median value and was chosen as cut-off for distinguishing slower from faster growing tumors within the G1 category.

**Figure 2:**
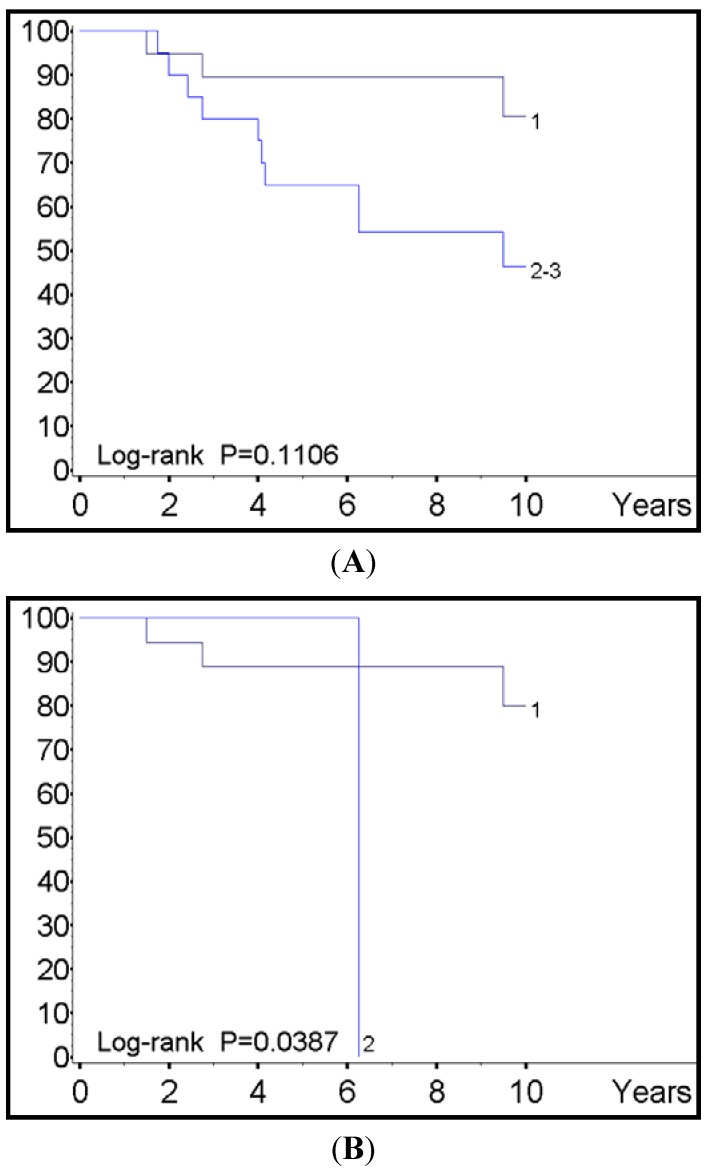
(**A**) SDHB immunostining intensity in primary tumors; (**B**) SDHB immunostining intensity in metastatic sites.

Survival analysis showed that the SDHB intensity but not the SDHB percentage of tumors cells impacted inversely on the patients’ prognosis, with marginal poorer prognosis being observed in individuals loosing SDHB immunoreactivity when considered as a whole (*p* = 0.1106) and significant shorter survival in the subset of metastatic diseases (*p* = 0.0387) (Figure 2). Other variables correlating with reduced survival included the lack of transplant treatment (*p* = 0.0094) and CgA levels >200 (*p* = 0.001). Although proliferative activity by means of Ki-67 labeling index was not a prognostic factor in this subset of patients, it was however marginally related to survival in metastases rather than primary tumors (*p* = 0.086). Multivariate analysis according to Cox’s model let emerge CgA level but not SDHB immunostaining intensity as an independent factor of survival (Table 4).

**Table 4:** Multivariate analysis in 31 nontransplanted patients.

	HR (95% CI)	*p* Value
**CgA level**		
<200	1.00	
>201	8.22 (1.02–66.4)	0.048
**SHDB intensity**		
1	1.00	
2–3	1.38 (0.49–3.91)	0.55

Interesting findings of our study were that SDHB expression correlated with the tumor cell differentiation and malignant potential of G1 INETs, and that the percentage of immunoreactive cells was associated with the staining intensity (Table 2). As a matter of fact the higher was the loss of SDHB immunoreactivity, the higher the proliferative activity (Table 3), the higher the likelihood of facing with metastatic sites (Table 3), and the shorter the survival (Figure 2a,b). Accordingly, SDHB was likely to behave as tumor suppressor gene in this category of neuroendocrine tumors, in that the lack of this protein was associated with parameters of clinical aggressiveness in metastatic tumors and reduced life expectation.

Although the switch from respiration to glycolysis in tumor cells has often been considered a consequence rather than a cause of cancer [45,46], the discovery that germline, inherited mutations in the genes encoding SDH enzyme subunits may cause paragangliomas and phaeochromocytomas [17,19,45,46,47], whether hereditary or sporadic, has however revolutionized this assumption [45,46]. Interestingly, all SDH subunits have no cytosolic counterpart unlike most Krebs cycle enzymes, but are imported into the mitochondria where they are modified, folded and assembled. Hence, they are able to deeply affect the capability of producing energy, whenever these subunits are downregulated in their expression. In our investigation, we observed that metastatic sites showed loss of SDHB expression along with a higher Ki-67 LI thereby supporting the contention that the development of anaerobic metabolism mechanisms could favor an increase of clinical aggressiveness.

In our paper, we provide evidence for the first time that the assessment of SDHB immunoreactivity in well-differentiated INETs may identify a subset of tumors characterized by reduced life expectation, which are worth treating more aggressively with multimodality therapy. Moreover, this paper challenges the common credence that G1 neuroendocrine tumors of the ileum are uniformly poorly aggressive tumors, while they are likely to be a heterogeneous tumor group harboring different lesions with different degrees of malignancy. The loss of function of SDHB as indicated by its relevant down-expression might be responsible for a pseudo-hypoxic drive via succinate-induced glycolysis and HIF stabilization in normoxic conditions [48], so favoring angiogenesis, tumor growth and progression of malignancy [48]. Recent evidences have corroborated the notion of a possible role for succinate accumulation due to SDHB activity loss also in epigenetic changes of chromatin via the histone H3 methylation in succinate-accumulating tumor cells [48]. Further investigation dealing with somatic mutation analysis of SDHB gene is currently in progress in our laboratory to better clarify additional molecular mechanisms underlying the loss of SDHB expression in this subset of INETs patients.

## 3. Experimental Section

### 3.1. Patients

Ileal neuroendocrine tumors from 31 patients (78% males and 22% females, median age 55.5 years, range 19 to 75 years) were retrieved from the archives of the Pathology Department of the National Cancer Institute of Milan. These cases had been surgically treated from 1992 to 2007 at the Department of Surgery of the same Institution. All INETs were G1 neuroendocrine tumors according to WHO/AJCC/ENET criterio for tumor grading (Ki-67 labeling index ≤2%). According to clinical and laboratory findings, two tumor groups were identified: FT group was defined by the occurrence of a compatible clinical syndrome associated with serum elevation and immunohistochemical detection of the relevant hormones, and NFT group by the absence of both clinical symptoms and serum elevation of hormones, regardless of the presence of immunostaining for any hormones [49,50,51,52,53,54,55,56,57,58,59]. All cases were subjected to serum and immunohistochemical assessment for CgA, synapthophisin, serotonin, and somatostatin receptor type 2A. Most of patients underwent surgical primary resection and all of them presented with distant synchronous liver metastases treated with nodule excision in 25 patients and liver transplant in 6 patients according to the so-called Milan criteria [60]. In the patients undergoing liver transplant, three clinical subgroups were considered according to the amount of liver involvement as assessed by surgical staging or CT scan: tumor load <25% (H1), between 25% and 50% (H2), and over 50% (H3) [61]. Clinicopathological data on the INETs under evaluation are shown in Table 1.

### 3.2. Tumors Specimens, Immunohistochemical Methods and Scoring of Data

The diagnosis of INETs was established by means of the last WHO classification [49,62]. All surgical samples (19 primary tumors, 9 metastases and 11 combined primary and metastatic lesions) had been fixed in 10% buffered formaldehyde solution and embedded in paraffin. To minimize the intratumoral variability because of sampling process, the entire tumor was immunostained if the lesion was up to 2 cm in diameter or at least two representative tissue blocks were immnnostained if the lesion was larger than 2 cm in diameter. Four μm-thick paraffin sections were reacted with monoclonal antibodies against CgA, synaptophysin, serotonin, Ki-67 antigen and SDHB [49] and processed according to previously refined immunohistochemical methods. Internal and external controls were used for all markers as appropriate.

In order to minimize variability in the slide assessment when trying punctual percentages, immunohistochemistry results for SDHB were rendered semiquantitatively on a scale from 1+ to 4+, taking into account a granular labeling product in the cytoplasm. One-plus tumors showed immunoreactivity in up to 25% neoplastic cells, 2+ cases in 26–50% neoplastic cells, 3+ cases in 51–75% neoplastic cells, 4+ cases in 76–100% neoplastic cells. Moreover, the immunostaining intensity was indicated as low (1+), if fainter than that seen in internal controls, or strong (3+), if more intense than the normal internal controls that was in turn indicated as being 2+. As SDHB is ubiquitous in normal cells, internal controls included any type of non-neoplastic cells, whether epithelial or mesenchymal.

### 3.3. Statistical Analysis

Associations of categorical variables were evaluated by Fisher’s exact *t*-test, test for trend or chi-square test. Survival estimates were calculated with Kaplan-Maier’s method and compared by Cox-Mantel’s log rank test. The comparative importance of explanatory variables on survival time was evaluated by means of Cox’s proportional hazard regression model. All the analyses were performed using the SAS statistical software (SAS Institute, Inc., Cary, NC, USA). All p-values were based on two-sided testing.

## 4. Conclusions

Our study provides the first evidence of a down-regulation of SDHB in well-differentiated INETs as likely mechanism contributing to the development of a subset of biologically more aggressive tumors as heralded by increased proliferative activity and reduced survival. Further investigation is currently in progress in our laboratory on a larger cohort of INETs patients, as well as in other types of neuroendocrine tumors, in order to confirm and expand these preliminary data.
